# The clinical significance of FAM19A4 methylation in high-risk HPV-positive cervical samples for the detection of cervical (pre)cancer in Chinese women

**DOI:** 10.1186/s12885-018-4877-5

**Published:** 2018-11-29

**Authors:** Qiaowen Bu, Sanfeng Wang, Jian Ma, Xiangcheng Zhou, Guiying Hu, Hua Deng, Xiaoli Sun, Xiaoshan Hong, Hengying Wu, Liang Zhang, Xiping Luo

**Affiliations:** 1 Department of Gynecology, Guangdong Women and Children Hospital, 521 Xing Nan Road, Guangzhou, 511400 Guangdong Province China; 2 Translational medicine center, Guangdong Women and Children Hospital, 521 Xing Nan Road, Guangzhou, 511400 Guangdong Province China; 3 Guangdong Women and Children Hospital of Guangzhou Medical University, Guangzhou, China

**Keywords:** Cervical cancer, HPV genotyping, Cytology, FAM19A4, DNA methylation, Quantitative PCR (qPCR)

## Abstract

**Background:**

To explore the diagnostic value of FAM19A4 methylation in high-risk human papilloma virus (hrHPV)-positive cervical samples from Chinese women for estimating cervical cancer or its precancerous lesions.

**Methods:**

Cervical samples from 215 women infected with high-risk HPV were collected by smear testing. We purposely chose 61 patients with cervical cancer, 57 with high-grade squamous intraepithelial lesions (HSIL), 31 with low-grade squamous intraepithelial lesions (LSIL), and 66 without cervical intraepithelial neoplasia (CIN) after histological confirmation. Taqman probe-based quantitative PCR (qPCR) was utilized to detect the methylation status of FAM19A4 in the cervical samples and further evaluate the use of this gene in the diagnosis of cervical cancer.

**Results:**

(1) An increasing level of FAM19A4 methylation was detected with increasing progression of cervical lesions, with methylation rates of 10.61%(7/66), 35.48%(11/31), 56.14%(32/57) and 93.44%(57/61) in no CIN, LSIL, HSIL and cervical carcinoma samples respectively. (2) In all hrHPV-positive samples, the levels of FAM19A4 methylation in HPV16/18 groups were higher than that in 12 other hrHPV groups (*P* < 0.05), but there was no significant difference between two groups after grouping cervical lesions into cervical cancer, HSIL, LSIL and no CIN groups (P>0.05). (3)There were no significant differences of FAM19A4 methylation in different clinicopathological parameters of cervical cancer. (4) Though the sensitivity of FAM19A4 methylation test was inferior to that of cytology and FAM19A4 combining with HPV16/18 genotyping, but showed the best specificity with 81.44% both for detection HSIL alone and ≥ HSIL, with favorable youden index (YI) and area under curve (AUC).

**Conclusion:**

FAM19A4 is a specific biomarker of cancerous lesions of the cervix. FAM19A4 methylation analysis may serve as an auxiliary screening method for diagnosis of cervical (pre)cancer. However, in consideration of the limitations of this retrospective study, prospective population-based studies are necessary for further confirmation of the diagnostic value of FAM19A4 methylation for detection of cervical (pre)cancer in Chinese women.

**Electronic supplementary material:**

The online version of this article (10.1186/s12885-018-4877-5) contains supplementary material, which is available to authorized users.

## Background

Cervical cancer is the third largest malignant tumor type suffered by women the world over, ranking fourth in cancer-related deaths of women each year [1]. The latest statistics show that there are 98,900 new cases and 30,500 deaths per year in China, and 12,900 new cases and 4100 deaths in the United States of America [2, 3]. A lack of early screening method in developing countries leads to the incidence and mortality of cervical cancer being greater than in developed countries. 94% of cervical cancer results from persistent infection with hrHPV [4, 5]. However, it is a long process, which can last 15–30 years from initial hrHPV infection to cervical cancer [6]. More than 80% of women become infected with HPV during their lifetime, 90% of which will be effectively cleared by their immune system, with 10% suffering persistent infection, and 1% progressing to cervical cancer [4]. Therefore, further screening methods are needed to identify which hrHPV-positive patient has a higher risk of developing cervical cancer or precancerous lesion. However, there is not an effective method to screen for cervical (pre)cancer. For example, the sensitivity and specificity of HPV16/18 genotyping in the identification of ≥CINII lesions are low with only 58.9% and 58.2%, respectively [7]. Although the cytological test (threshold borderline ASCUS) has a higher specificity, but still misses 30% of ≥CINII lesions [8]. Recent studies have found that detecting methylation of related biomarkers not only maintains the sensitivity but also increases the specificity and helps to identify cervical cancer and its precancerous lesions [9, 10]. Thus, it is essential to explore more specific cancer biomarkers to identify cervical (pre)cancer.

Previous studies suggested that persistent infection with hrHPV was not sufficient to cause immortalization and transformation of cervical epithelial cells, and epigenetic changes played an important role in developing of cervical cancer [11]. Most studies showed that DNA methylation is one of the most common molecular mechanisms apparent in cervical cancer. Abnormal methylation of tumor suppressor gene promoters was closely related to the occurrence and development of cervical cancer, which could be detected in 70–100% of cervical cancer and 30–80% of cervical precancerous lesions [12]. A recent study reported that several potential biomarkers, such as PAX1, SOX1, ZNF582, and NKX6–1, were of value as a marker for the detection of cervical (pre)cancer among hrHPV-positive women [13]. Furthermore, methylation levels of some genes (CADM1, ZSCAN1, ST6GALNAC5, ANKRD18CP, CDH6, GFRA1, GATA4, KCNIP4, LHX8 and FAM19A4) have been shown to increase with the severity of the underlying histological lesion in cervical scrapes [7, 14–19].

FAM19A4 (family with sequence similarity 19 (chemokine (C–C motif)-like) member A4) is a member of the TAFA gene family that encodes small molecule proteins. The encoded protein contains a conserved cysteine residue in a fixed position and is associated with stress and inflammation. FAM19A4, as a ligand of formyl peptide receptor 1(FPR1), can promote phagocytosis and increase reactive oxygen species release by macrophages. It is typically upregulated in lipopolysaccharide-stimulated monocytes and macrophages [20, 21] .In recent years, some studies have revealed that FAM19A4 methylation has a close relationship with cervical cancer and is a putative cervical cancer biomarker and an effective triage method for hrHPV-positive women in cervical screening [7, 14, 15, 22–24]. However, the application value of FAM19A4 methylation in triage of hrHPV-positive women in China has not yet been studied yet. Therefore, we first conduct a retrospective study to investigate the value of FAM19A4 methylation in diagnosis of cervical cancer and its precancerous lesions, which will lay the foundation for further prospective studies on FAM19A4 methylation in triage of hrHPV-positive women in China.

## Methods

### Study participants and specimens collection

**Fig. 1:**
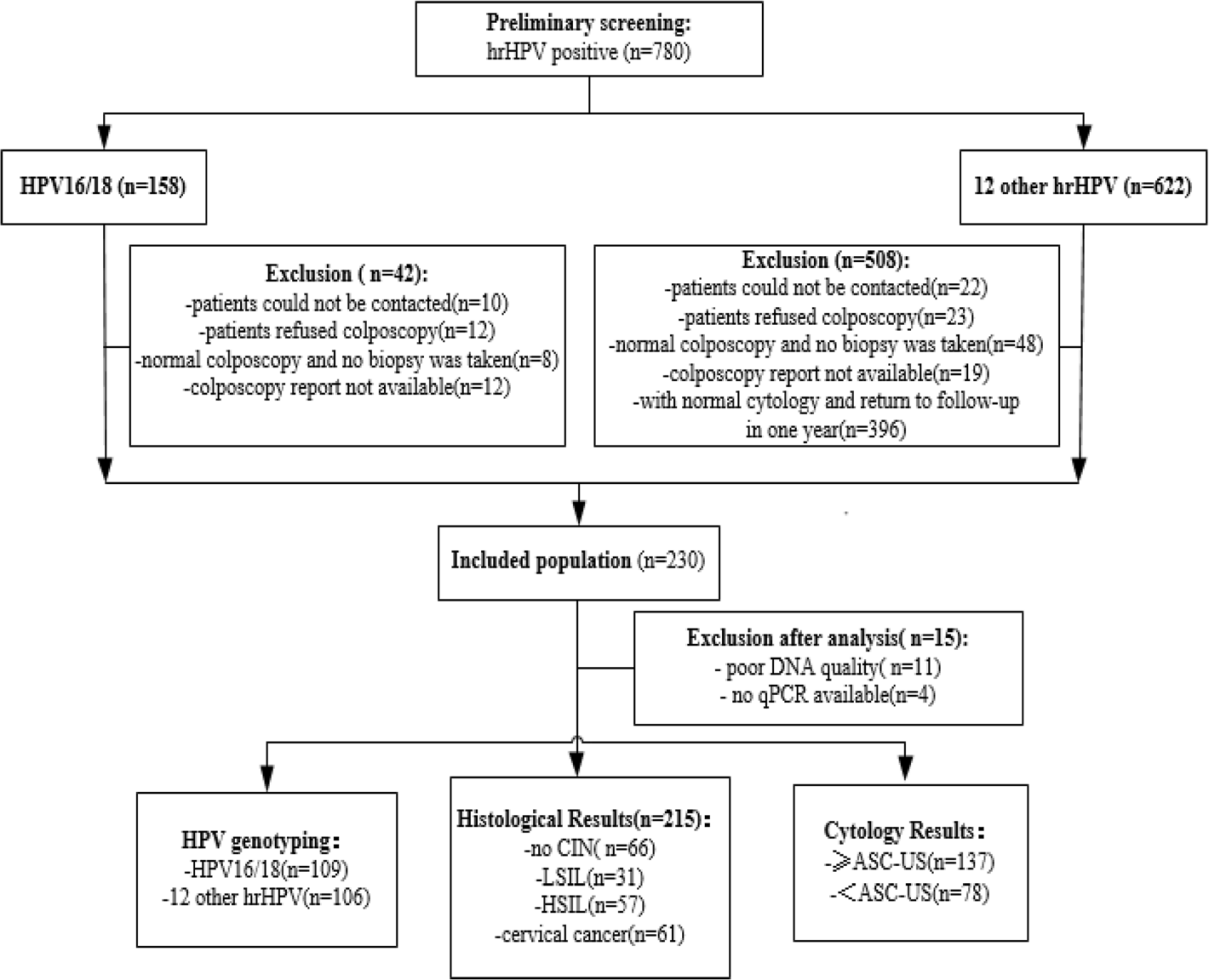
Flowchart of study population. 12 other hrHPV refer to HPV31, 33, 35, 39, 45 51, 52, 56, 58, 59, 66 and 68 infection

The screening flowchart was shown in Fig. 1. 215 patients enrolled in HPV genotyping testing at *Guangdong Women and Children Hospital* between November 2016 and November 2017 were purposely selected. Cervical samples were collected by experienced gynecologists using a cervical brush and qPCR was used to detect for 14 hrHPV genotypes (including HPV16, 18, 31, 33, 35, 39, 45 51, 52, 56, 58, 59, 66 and 68) according to the manufacturer’s instructions (Kaipu company). Patients with cytology ≥atypical squamous cells of unknown significance (ASCUS) + hrHPV positive or HPV16/18 positive or clinical examination of suspected abnormality or suspected history and signs (eg: contact bleeding, abnormal vaginal secretions, abnormal vaginal bleeding and abnormal cervical morphology, ect)were referred for colposcopy. Biopsies and/or endocervical curettage were taken from abnormal cervical areas during colposcopic examinations [25].Of the 215 patients that underwent colposcopy and subsequent biopsy, 61 patients were biopsy-confirmed with cervical cancer, 57 with HSIL, 31 with LSIL, and 66 were considered no CIN by two or more professional pathologists. The study was approved by the local ethics committee (reference number: 201701005) and all participants gave informed consent before specimens collection according to institutional guidelines. Exclusion criteria included current pregnancy or lactation and current or previous history of cancer [22].

**Fig. 2:**
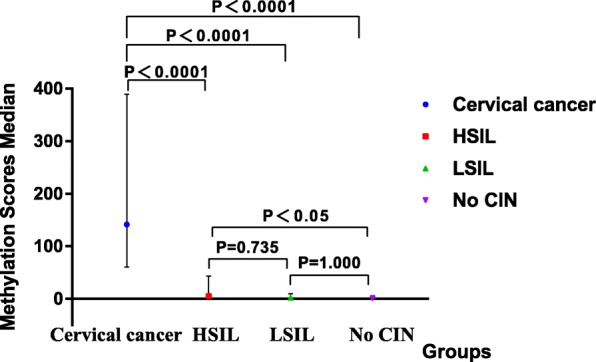
Methylation scores median of different cervical lesions. In this box-and-whisker plot, the boxes represent median values; the upper and lower lines outside the boxes represent the 25th and 75th percentiles, respectively

### DNA extraction, DNA bisulfite modification and MSP-qPCR

Genomic DNA from hrHPV cervical samples was extracted using the TIANamp Genomic DNA Extraction Kit (Tiangen, Beijing) according to the manufacturer’s instructions. DNA concentrations were measured using a Quawell Q5000 UV spectrophotometer.

The extracted DNA (template DNA concentrations of 500 ng) was bisulfite-converted using the EZ DNA Methylation-Direct ™ kit (Zymo Research, Irvine, USA) according to the manufacturer’s recommendations. The bisulfite-modified DNA was immediately used for qPCR.

FAM19A4 methylation in hrHPV cervical samples was performed by qPCR using the ABI 7500 Real Time Fluorescence Quantitative PCR System (Life Tech, America). DNA from cervical cancer and normal peripheral blood, and double distilled water (ddH_2_O) were used as the positive control, negative control, and blank control, respectively. Amplification reactions were performed in triplicate in a total volume of 20 μl consisting of 10 μl 2 × Premix Type reagent; 5.2 μl ddH2O; 1 μl bisulfite-converted DNA sample; 0.2 μl 50 × Rox Reference Dye II reagent; 0.5 μl of each forward and reverse primer of target gene (FAM19A4) and the house keeping genes beta actin (ACTB); and 0.8 μl of each probe of FAM19A4 and ACTB.

The qPCR primer and probe used in this study were based on Steenbergen’s study [14]. But some minor adjustments at certain sites had been modified according to experimental situation, in order to achieve better amplification efficiency. The following primer sequences were used for the FAM19A4 gene: Forward primer: 5’-CGGGCGGTTCGGTTAATT-3’ Reverse primer 5’-AAAACGACGCGCAACTAAC-3′(101 bp). The following primers sequences were used for ACTB internal reference gene: Forward primer: 5′-TGGTGATGGAGGAGGTTTAGTAAGT-3’ Reverse primer 5’-AACCAATAAAACCTACTCCTCCCTTAA -3′(133 bp).

The probe sequences of FAM19A4 and ACTB were as follows: FAM-CCGAACCCAACTAACGCGCTAACCAA-BHQ1 and HEX-ACCACCACCCAACACACAATAACAA -ACACA-BHQ1. The PCR program consisted of the following steps: hot start activation 3 min 95 °C; 40 cycles of denaturation 15 s 95 °C, annealing and extension 1 min 60 °C. Amplification results were determined by collecting the fluorescent signal to obtain the circulating threshold (CT value) and the amplification curve of ACTB and FAM19A4 (Additional file 1). Samples with a CT > 40 for FAM19A4 were considered to represent a negative test result. All samples had a CT value for ACTB < 32 to ensure good sample quality. FAM19A4 methylation scores were calculated using the following formula: 2^[Ct (ACTB) -Ct (FAM19A4)]^ × 100 [15].

### Statistical analysis

Statistical analyses were performed using the IBM SPSS Statistics Version 23 (IBM Corp, Armonk, NY, USA), and all statistical analyses were two-sided. Continuous variables of abnormal distribution were represented by Median($\mathrm{P}\frac{1}{4}-\mathrm{P}\frac{3}{4}$). Kruskal-Wallis H Test and Mann-Whitney U Test were used for univariate analysis of the continuous variables to calculate the differences of methylation scores among groups. Chi squared test was used for categorical variables. Cochran-Armitage trend test was used to analyze the linear correlation between cervical lesions and FAM19A4 methylation. Spearman association analysis was used to analyze the relationship between FAM19A4 methylation and HPV genotyping. Logistic regression was used to analyze the influence of several factors on FAM19A4 methylation: the ages of the participants, the cytology results(≥ASC-US or<ASC-US) and the HPV genotyping(HPV16/18 or 12 other hrHPV). A *p*-value of < 0.05 was considered to be statistically significant for the above statistical methods.

## Results

### Comparison of FAM19A4 methylation in hrHPV–positive samples with differing severity of cervical lesions

The median methylation scores of the four groups tested were 0.74(0.002–5.70), 2.27(0.23–9.79), 5.36(0.48–43.70) and 141.42(59.95–389.23), in no CIN, LSIL, HSIL and cervical cancer samples respectively, with significant statistical differences among four groups(*P* < 0.05). In pairwise comparisons between groups, the median methylation scores of FAM19A4 in the HSIL, LSIL, and no CIN groups were all lower when compared with the cervical cancer group (P < 0.05). And the median methylation scores of HSIL group were also higher than that of no CIN group (P < 0.05) (Fig. 2) (Table 1).

**Table 1:** Comparison of FAM19A4 methylation in hrHPV–positive samples with differing severity of cervical lesions

Results	Median Methylation	Methylation Rates	cOR	aOR
Category	Scores($\mathrm{P}\frac{1}{4}-\mathrm{P}\frac{3}{4}$)	(%)	(95%CI)	(95%CI)
Cervical cancer	141.42(59.95–389.23)	93.44(57/61)	120.11(33.35–432.53)	59.171(14.911–234.801)
HSIL ^a^	5.36(0.48–43.70)	56.14(32/57)	10.79(4.21–27.68)	9.061(3.208–25.596)
LSIL ^b^	2.27(0.23–9.79)	35.48(11/31)	4.64(1.58–13.58)	4.862(1.542–15.324)
No CIN ^c,d^	0.74(0.002–5.70)	10.61(7/66)	1.00	1.00

The cOR was calculated by multinomial regression analysis, using four different types of cervical lesions as the dependent variables, FAM19A4 methylation(positive or negative) as an independent variable

The aOR was calculated by multinomial regression analysis, correcting other factors of ages, HPV genotyping(HPV16/18 or non-HPV16/18) and cytology(<ASC-US or ≥ ASC-US), and also used four different types of cervical lesions as the dependent variables, FAM19A4 methylation(positive or negative) as an independent variable

*P* percentiles, *CI* confidence interval, *cOR* crude odds ratio, *aOR* adjusted odds ratio

Methylation scores were used to calculate the statistical difference

^a^: compared with cervical cancer, H = 85.55, P < 0.0001, Kruskal-Wallis H Test

^b^: compared with cervical cancer, H = 116.21, *P* < 0.0001, Kruskal-Wallis H Test

^c^: compared with cervical cancer, H = 122.11, P < 0.0001, Kruskal-Wallis H Test

^d^: compared with HSIL, H = 36.551, *P* < 0.05, Kruskal-Wallis H Test

Cochran-Armitage trend tests showed that there was a linear trend between cervical lesions and FAM19A4 methylation. A highly significant trend for increasing FAM19A4 methylation with increasing histological severity (Cochran-Armitage trend test, P < 0.05), which were 10.61% (7/66), 35.48%(11/31), 56.14%(32/57), and 93.44% (57/61) in the no CIN, LSIL, HSIL, and cervical cancer groups respectively.

Among the four groups with differing severity of cervical lesions, the methylation of FAM19A4 was most easily detected in cervical cancer, followed by HSIL, LSIL and no CIN groups. Compared with the group of no CIN, the crude odds ratio(cOR) of FAM19A4 methylation in cervical cancer, HSIL and LSIL groups were 120.11 (95%CI:33.35–432.53),10.79 (95%CI:4.21–27.68)and 4.64 (95%CI: 1.58–13.58). After correcting other factors of ages, HPV genotyping (HPV16/18 or 12 other hrHPV) and cytology (<ASC-US or ≥ ASC-US), the adjusted OR(aOR) of FAM19A4 methylation also increased as the disease severity increased (Table 1).

### Relationship between FAM19A4 methylation and hrHPV genotyping in hrHPV-positive samples

The differences of FAM19A4 methylation between HPV16/18 groups and 12 other hrHPV groups were highlighted in Table 2. Of the 215 patients with hrHPV infection, there was a significant difference of FAM19A4 methylation between samples positive for HPV16/18 and 12 other hrHPV(*P* < 0.05). The relationship between FAM19A4 and hrHPV genotyping was also analyzed by Spearman association analysis, suggesting that there was a positive correlation between HPV16/18 infection and FAM19A4 methylation in all hrHPV-positive samples (*r* = 0.386, P < 0.05).

**Table 2:** Relationship between FAM19A4 methylation and hrHPV genotyping in hrHPV-positive samples

	HPV genotype	Median Methylation	Methylation Rates	U	P
		Scores($\mathrm{P}\frac{1}{4}-\mathrm{P}\frac{3}{4}$)	(%)		
All samples	HPV16/18	32.88(2.91–5129.12)	68.81(75/109)	3268.50	3.783 × 10^–8*^
	12 other hrHPV ^a^	2.05(0.22–12.10)	30.19(32/106)		
Cervical cancer	HPV16/18	141.42(60.63–363.07)	93.88(46/49)	289.50	0.935^**^
	12 other hrHPV	141.34(30.84–842.89)	91.67(11/12)		
HSIL	HPV16/18	14.65(1.67–56.09)	64.29(18/28)	305.00	0.107^**^
	12 other hrHPV	4.35(0.36–36.85)	48.28(14/29)		
LSIL	HPV16/18	3.20(0.90–21.98)	40.00(4/10)	91.00	0.574^**^
	12 other hrHPV	1.67(1.20–18.35)	33.33(7/21)		
No CIN	HPV16/18	1.83(0.50 × 10^−2^-24.21)	31.82(7/22)	347.00	0.062^**^
	12 other hrHPV	0.66(0.19 × 10^−2^-2.13)	0.00(0/44)		

^a^12 other hrHPV refer to HPV31, 33, 35, 39, 45 51, 52, 56, 58, 59, 66 and 68 infection

*P<0.05,Mann-Whitney U Test, methylation scores were used to calculate the statistical difference

**P>0.05,Mann-Whitney U Test, methylation scores were used to calculate the statistical difference

In addition, after grouping cervical lesions, what stood out was that no significant increase in HPV16/18 groups was found compared with 12 other hrHPV-positive group in different cervical lesions (P>0.05) (Table 2).

### Relationship between clinicopathological features of cervical cancer and FAM19A4 methylation in hrHPV-positive samples

The average age of the 61 patients with cervical cancer was 47.98 years. There was no significant difference in FAM19A4 methylation associated with ages, pathological types, clinical stage (FIGO, 2009), tumor size, lymph node metastasis, or HPV infection types (*P* > 0.05) (Table 3).

**Table 3:** Relationship between FAM19A4 methylation and clinicopathological parameters in cervical cancer

Parameters	Median Methylation	Methylation Rates	U	P^*^
	Scores($\mathrm{P}\frac{1}{4}-\mathrm{P}\frac{3}{4}$)	(%)		
Age				
< 48	144.14(49.89–387.46)	90.63(29/32)	445.00	0.784
≥48	140.65(62.20–466.41)	96.55(28/29)		
Histology				
SCC	145.37(62.54–450.44)	94.44(51/54)	144.00	0.321
AC/ASC	137.83(8.38–207.24)	85.71(6/7)		
Stage				
IA or IB1	153.17(67.84–387.46)	91.67(33/36)	409.00	0.548
IB2 or above	97.78(51.19–562.08)	96.00(24/25)		
Size				
≤4 cm	141.42(62.20–389.23)	93.88(46/49)	279.00	0.786
> 4 cm	123.54(23.42–807.25)	91.67(11/12)		
Lymph node metastasis				
yes	167.28(77.43–604.86)	100(10/10)	227.00	0.585
no	140.65(55.49–366.61)	92.16(47/51)		

*SCC* squamous cell carcinoma, *AC* adenocarcinoma, *ASC* adenosquamous carcinoma

*P>0.05,Mann-Whitney U Test, methylation scores were used to calculate the statistical difference

### Clinical performance indicators of FAM19A4 methylation, cytology and HPV16/18 genotyping for detection HSIL alone and HSIL or cervical cancer (≥HSIL)

**Fig. 3:**
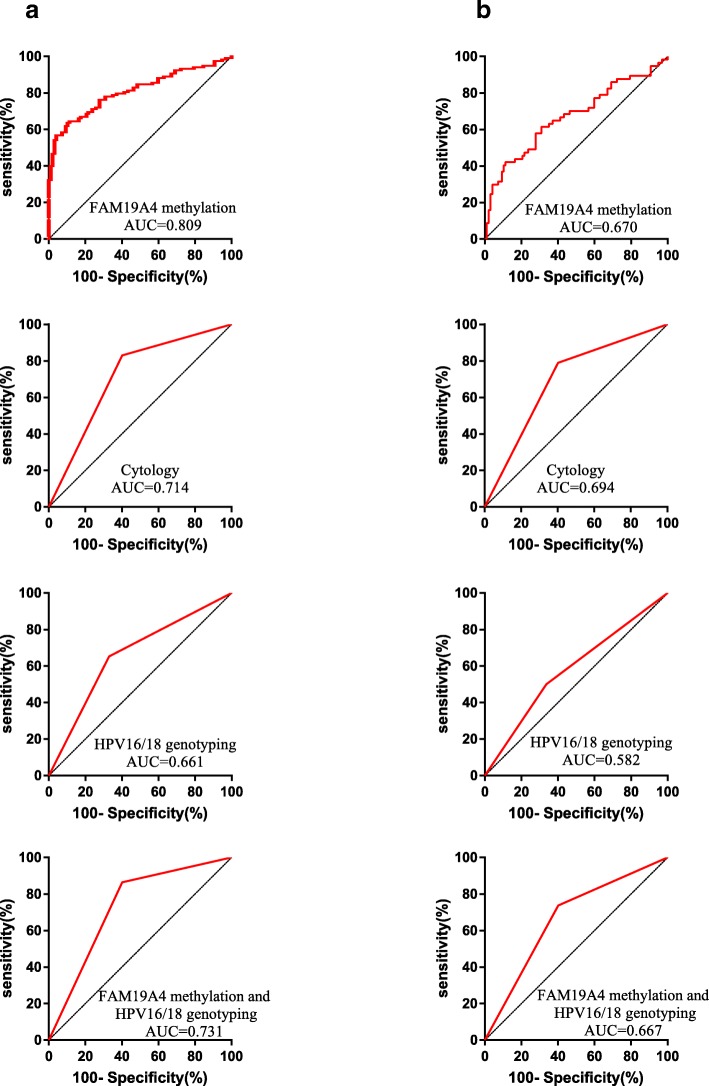
The diagnostic power of FAM19A4 methylation, cytology, HPV16/18 genotyping and the combination of FAM19A4 methylation and HPV16/18 genotyping. (**a**) Power of FAM19A4 methylation, cytology, HPV16/18 genotyping and the combination of FAM19A4 methylation and HPV16/18 genotyping in differentiating ≥HSIL patients from ≤LSIL patients. (**b**) Power of FAM19A4 methylation, cytology, HPV16/18 genotyping and the combination of FAM19A4 methylation and HPV16/18 genotyping in differentiating HSIL patients from ≤LSIL patients

For detecting HSIL alone, FAM19A4 methylation had a lower sensitivity than cytological test and FAM19A4 methylation combining with HPV16/18 genotyping test, whereas its specificity was significantly higher than any other test(P<0.05). Although the sensitivity of FAM19A4 methylation analysis for the detection HSIL alone was higher compared with HPV16/18 genotyping (56.14% vs 49.12%), the difference was not statistically significant (P>0.05). ROC curve analysis also showed an AUC of 0.67(P<0.05) of FAM19A4 methylation test in detecting HSIL alone, slightly lower than cytological test (0.67 vs 0.69), but similar as the test of FAM19A4 methylation combining with HPV16/18 genotyping (0.67 vs 0.67), and higher than HPV16/18 genotyping (0.67 vs 0.58) (Fig. 3). In addition, FAM19A4 methylation analysis showed an advantageous YI in detecting HSIL alone (Table 4).

**Table 4:** Power of FAM19A4 methylation, cytology and HPV16/18 genotyping for detection HSIL alone and ≥ HSIL

Triage	Endpoint	AUC (95%CI)	Sensitivity (%(95%CI))	Specificity (%(95%CI))	YI (%)
FAM19A4	HSIL alone	0.67(0.58–0.76)	56.14(43.26–69.02)	81.44 (73.71–89.18)	37.58
	≥HSIL	0.81(0.75–0.87)	75.42(67.66–83.19)	81.44 (73.71–89.18)	56.86
Cytology	HSIL alone	0.69(0.61–0.78)	78.95(68.36–89.53)^a^	59.79(50.04–69.55) ^c^	38.74
	≥HSIL	0.71(0.64–0.79)	83.05(76.28–89.82) **	59.79(50.04–69.55)^d^	42.84
HPV16/18 genotyping	HSIL alone	0.58(0.49–0.68) *	49.12(36.14–62.10) **	67.0(57.65–76.37) ^c^	16.13
	≥HSIL	0.66(0.59–0.74)	65.25(56.66–73.85) **	67.01(57.65–76.37)^d^	32.26
FAM19A4 and HPV16/18	HSIL alone	0.67(0.58–0.76)	73.68(62.25–85.12) ^a^	59.79(50.04–69.55) ^c^	33.48
	≥HSIL	0.73(0.66–0.80)	86.44(80.26–92.62)^b^	59.79(50.04–69.55) ^d^	46.23

*AUC* area under curve, *YI* youden index

*P>0.05

**compared with the sensitivity of FAM19A4 in group of HSIL alone or ≥ HSIL, P>0.05

^a^: compared with the sensitivity of FAM19A4 in group of HSIL alone, *P* < 0.05

^b^: compared with the sensitivity of FAM19A4 in group of ≥HSIL, P<0.05

^c^: compared with the specificity of FAM19A4 in group of HSIL alone,P<0.05

^d^: compared with the specificity of FAM19A4 in group of ≥HSIL,P<0.05

As for detecting ≥HSIL, the sensitivity of FAM19A4 methylation test was inferior to that of FAM19A4 combining with HPV16/18 genotyping, but showed the best specificity with 81.44% for the detection ≥HSIL, with the most favorable YI.

ROC curve analysis also showed an AUC of 0.81 of FAM19A4 methylation to discriminate ≥HSIL from ≤LSIL, which was the best of all the tests.

## Discussion

To date, more than 100 genes have been found to cause methylation and gene silencing in cervical cancer and can be used as potential biomarkers for predicting cervical cancer [8]. Our previous study revealed that detection of FAM19A4 methylation was able to effectively distinguish cervical cancer and healthy cervical tissue (96.8% vs 8.7%,*P* < 0.05) at formalin-fixed and paraffin -embedded (FFPE) levels, suggesting that FAM19A4 could be a promising biomarker of cervical carcinoma, which is consistent with Steenbergen’s study [14], showing a significant difference in cervical squamous cell carcinoma and normal cervical tissue (91% vs 5%, P < 0.05). However, tissue samples were not widely used in clinical screening diagnosis due to their acquisition being invasive; but cervical exfoliative scrapes were easy to obtain non-invasively, and more suitable for clinical diagnosis and follow-up, with greater clinical value. In line with De Strooper and Steenbergen’s reporting on FAM19A4 methylation levels increasing with cervical lesion severity [14, 15], the present study also demonstrated that FAM19A4 methylation increased with increasing progression of cervical lesions. This study further calculated the OR of FAM19A4 methylation in cervical lesions with different severity compared with no CIN group, indicating that FAM19A4 methylation was closely correlated to cervical cancer and its precancerous lesions.

As a highly effective biomarker, FAM19A4 was reported to be a promising triage tool for hrHPV-positive women [7, 15, 22, 23]. De Strooper et al. [15] evaluated that FAM19A4 methylation was detected in all cervical cancer and CINII/III lesions with HPV infection lasting ≥5 years (advanced CINII/III), but only 82.8% of cervical cancer and 86.4% of advanced CINII/III were detected by cytological tests, revealing FAM19A4 methylation could predict those patients with high risk of progression to cervical cancer and its precancerous lesion. A similar sensitivity of FAM19A4 methylation analysis compared to cytology (69.2% vs 63.5%) was also observed at a higher specificity for detecting ≥CINII lesions (83.7% vs 69.6%). Coincidentally, Luttmer et al. [7] also believed that FAM19A4 methylation for detection CINII/III lesions was not significantly inferior to the tests of cytology or HPV16/18 genotyping, but yielded an increased specificity more than 70%. This study also presented FAM19A4 methylation analysis had a better advantage than cytology and. HPV16/18 genotyping in detecting cervical (pre)cancer, which was concordant with the reported studies [7, 15]. Luttmer’s studies demonstrated that after combining HPV16/18 genotyping with FAM19A4 methylation, the sensitivity for detection ≥CINIII of FAM19A4 methylation was increased, but with an inferior specificity [7, 23]. This research also showed the similar clinical performance of FAM19A4 methylation. ROC analysis for the diagnostic power of FAM19A4 methylation yielded an AUC of 0.81 with 75.42% sensitivity and 81.44% specificity in differentiating patients with ≥HSIL lesions from those with ≤LSIL lesions. These results suggested that FAM19A4 is a valuable biochemical marker to detect cervical cancer and its precancerous lesions in hrHPV-positive women. The limitation was that the clinical performance indicators calculated in this study could not be used as a triage of hrHPV-positive women, while the diagnostic power of this study provided a preliminary research for further prospective cohort study of hrHPV-positive triage.

In order to improve the population rates of population-based screening, the utilization of self-sampled specimens played an indispensable role. Several studies have proved HPV self-sampled is an attractive tool for cervical screening [22, 23, 26, 27]. A recent study verified the specificity of FAM19A4 methylation analysis for detection of ≥CINII/III was higher in self-sampled lavage compared with that in physician-taken scrapes(81.3–82.8% vs 72.0–75.1%), with a non-inferior sensitivity [15]. In addition, FAM19A4 methylation analysis for detecting ≥CINIII presented a better clinical performance indicators than that of HPV16/18 genotyping in both lavage- and brush- self-sampled specimens. After combining FAM19A4 methylation HPV16/18 genotyping the sensitivity increased to more than 80%, yet at a cost of lower specificity for both sample types [23]. Although this study did not conduct a research of self-collected samples in Chinese women, on the basis of this study, it was of great help to further explore the comparison of FAM19A4 methylation for detecting cervical cancer and its precancerous lesion in different cervical exfoliative specimens.

In this study, FAM19A4 methylation differed significantly between samples infected with HPV16/18 or 12 other hrHPV in all samples. However, in each group of different cervical lesions, no group was detected differences of FAM19A4 methylation between HPV16/18 and 12 other hrHPV groups, which seemed to be the opposite of the above results. We found that the reason for this phenomenon was the levels of FAM19A4 methylation were mainly affected by severity of different cervical lesions, but not by HPV genotyping. Due to more than 70% of cervical cancer was caused by HPV16/18 [28], a higher proportion of cervical cancer was in HPV16/18 group, which led to FAM19A4 methylation differences between HPV16/18 and 12 other hrHPV groups from the all sample. Additionally, We calculated the statistical significance using the FAM19A4 methylation scores instead of the methylation rates considering the former is a relatively quantitative index, and found that the 7 samples in the no-CIN group had slight higher methylation scores than the cut-off value. Although there appeared to be a significant difference in methylation rates between HPV16/18 and 12 other hrHPV groups in no-CIN group (31.82% vs 0.00%), the lower methylation scores in HPV16/18 group resulted in no significant difference between the two groups. This situation also followed the aforementioned statement that the levels of FAM19A4 methylation increased with increasing progression of cervical lesions. In summary, whether HPV genotyping had a clear impact on the occurrence of methylation remained undetermined. Moreover, when explored the relationship between HPV genotyping and FAM19A4 methylation after grouping cervical lesions, the sample size of each group was relatively small, which might cause bias to some extent. Therefore, the follow-up studies required a larger sample size to verify, and further researches of mechanism were needed to investigate the relationship between HPV infection and FAM19A4 methylation.

This study first compared the differences in FAM19A4 methylation between different clinicopathological features of cervical cancer. Several meta-analysis reported that some genes (such as RASSF1A, CDH1, ESR1) methylation were not associated with tumor stage, which suggested these genes methylation might not play a substaintial role in the progression and prognosis of cervical cancer [29–31]. In this study, there were no statistically significant differences in FAM19A4 methylation between early cervical cancer (stage IA or IB1) and advanced cervical cancer (stage IB2 or above), indicating abnormal methylation of FAM19A4 was an early event in cervical cancer and might not be related to the severity of cervical cancer [32]. What’s more, the expression of FAM19A4 methylation was not significantly different in the presence or absence of lymph node metastasis, suggesting that FAM19A4 methylation was unlikely to play a crucial role in cancer invasion and metastasis. Yin et al. [33]found that the expression of STK31 methylation showed no differences in related clinicopathological features of cervical cancer(ages, histology, tumor size and lymph node metastasis), which also implied that gene methylation was related to the early occurrence of tumors, but not related to tumor invasion and metastasis.

Of the 61 cases cancerous samples in this study, 4 cases were diagnosed without FAM19A4 methylation. Among them, one tissue sample was unable to be obtained without surgery, and the other 3 cases all had hypermethylation detected in their corresponding FFPE tissues. The reason why cervical smear samples brought about false negative results might be due to poor extraction of pathological cells, resulting in inaccurate diagnosis. Of these 3 misdiagnosed cases, all were infected with HPV16/18 and cytological tests were normal, ASC-H and HSIL, respectively. These revealed that although FAM19A4 methylation had an excellent performance, there was still a risk of misdiagnosis. Combining FAM19A4 methylation with hrHPV and cytological tests can compensate for the deficiency of a single molecular diagnosis and reduce the misdiagnosis rates.

## Conclusions

In summary, this study found that FAM19A4 methylation occurred at an early stage in cervical cancer development. FAM19A4 methylation maybe serve as an auxiliary screening method for diagnosis of cervical (pre)cancer. However, prospective population-based studies are necessary for further confirmation of the diagnostic value of FAM19A4 methylation for detection of cervical (pre)cancer in Chinese women.

## Additional file

Additional file 1: Amplification curve of FAM19A4 and ACTB genes in cervical cancer, normal peripheral blood DNA, and ddH2O. (DOCX 42 kb)

## Abbreviations

ASCUS: Atypical squamous cells of unknown significance
AUC: Area under curve
CI: Confidence interval
CIN: Cervical intraepithelial neoplasia
DNMT: DNA methyltransferases
FAM19A4: Family with sequence similarity 19 (chemokine (C–C motif)-like) member A4
FFPE: Formalin-fixed and paraffin-embedded
HPV: Human papilloma virus
HSIL: High-grade squamous intraepithelial lesions
LSIL: Low-grade squamous intraepithelial lesions
OR: Odds ratio
qPCR: Taqman probe-based quantitative PCR
