# Supplementary material for: The clinical significance of FAM19A4 methylation in high-risk HPV-positive cervical samples for the detection of cervical (pre)cancer in Chinese women
Source: BMC Cancer. 2018 Nov 29;18:1182. doi: 10.1186/s12885-018-4877-5 (PMC6263049; doi:10.1186/s12885-018-4877-5)

**Additional file 1: Amplification curve of FAM19A4 and ACTB genes in cervical cancer, normal peripheral blood DNA, and ddH2O**


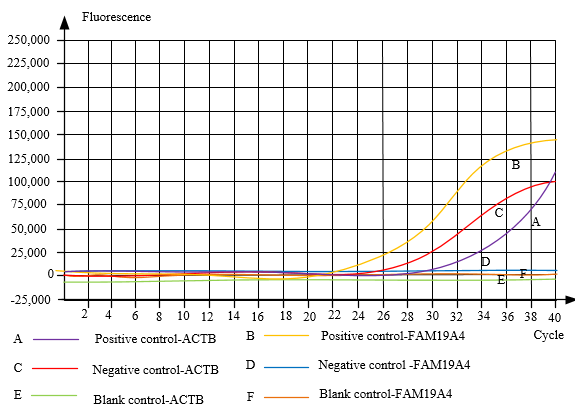

Supplement: Supplementary file 1 — Amplification curve of FAM19A4 and ACTB genes in cervical cancer, normal peripheral blood DNA, and ddH2O. (DOCX 42 kb) [file 12885_2018_4877_MOESM1_ESM.docx]
